# Diagnostic properties of the Frontal Assessment Battery (FAB) in Huntington’s disease

**DOI:** 10.3389/fpsyg.2022.1031871

**Published:** 2022-11-30

**Authors:** Federica Solca, Edoardo Nicolò Aiello, Simone Migliore, Silvia Torre, Laura Carelli, Roberta Ferrucci, Alberto Priori, Federico Verde, Nicola Ticozzi, Sabrina Maffi, Consuelo Ceccarelli, Ferdinando Squitieri, Vincenzo Silani, Andrea Ciammola, Barbara Poletti

**Affiliations:** ^1^Department of Neurology and Laboratory of Neuroscience, IRCCS Istituto Auxologico Italiano, Milan, Italy; ^2^PhD Program in Neuroscience, School of Medicine and Surgery, University of Milano-Bicocca, Monza, Italy; ^3^Huntington and Rare Diseases Unit, Fondazione IRCCS Casa Sollievo Della Sofferenza Research Hospital, San Giovanni Rotondo, Italy; ^4^Aldo Ravelli Center for Neurotechnology and Experimental Brain Therapeutics, Department of Health Sciences, International Medical School, University of Milan, Milan, Italy; ^5^ASST Santi Paolo e Carlo, San Paolo University Hospital, Milan, Italy; ^6^IRCCS Ca’ Granda Foundation Maggiore Policlinico Hospital, Milan, Italy; ^7^Department of Pathophysiology and Transplantation, Dino Ferrari Center, Università degli Studi di Milano, Milan, Italy; ^8^Italian League for Research on Huntington (LIRH) Foundation, Rome, Italy

**Keywords:** Huntington’s disease, cognitive screening, dysexecutive, diagnostics, psychometrics, frontal assessment battery

## Abstract

**Background:**

This study aimed at assessing the diagnostic properties of the Frontal Assessment Battery (FAB) as to its capability to (1) discriminate healthy controls (HCs) from patients with Huntington’s disease (HD) and (2) identify cognitive impairment in this population.

**Materials:**

Thirty-eight consecutive HD patients were compared to 73 HCs on the FAB. Patients further underwent the Montreal Cognitive Assessment (MoCA) and the Unified Huntington’s Disease Rating Scale (UHDRS). Receiver-operating characteristics (ROC) analyses were run to assess both intrinsic—i.e., sensitivity (Se) and specificity (Sp), and post-test diagnostics, positive and negative predictive values (PPV; NPV) and likelihood ratios (LR^+^; LR^–^), of the FAB both in a case–control setting and to identify, within the patient cohort, cognitive impairment (operationalized as a below-cut-off MoCA score). In patients, its diagnostic accuracy was also compared to that of the cognitive section of the UHDRS (UHDRS-II).

**Results:**

The FAB and UHDRS-II were completed by 100 and 89.5% of patients, respectively. The FAB showed optimal case–control discrimination accuracy (AUC = 0.86–0.88) and diagnostic properties (Se = 0.68–0.74; Sp = 0.88–0.9; PPV = 0.74–0.8; NPV = 0.84–0.87; LR^+^ = 5.6–7.68; LR^–^ = 0.36–0.29), performing even better (AUC = 0.9–0.91) at identifying cognitive impairment among patients (Se = 0.73–1; Sp = 0.86–0.71; PPV = 0.79–0.71; NPV = 0.82–1; LR^+^ =5.13–3.5; LR^–^ = 0.31–0) and comparably to the UHDRS-II (89% vs. 85% of accuracy, respectively; *p* = 0.46).

**Discussion:**

In HD patients, the FAB is highly feasible for cognitive screening aims, being also featured by optimal intrinsic/post-test diagnostics within both case-control and case-finding settings.

## Background

Cognitive impairment in Huntington’s disease (HD) patients entails detrimental impacts towards their prognosis (McAllister et al., 2021). Hence, its early detection *via* cognitive screeners is clinically crucial (Paulsen, 2011)—also in the view of planning *ad hoc*, either pharmacological (Dickey and La Spada, 2018) or non-pharmacological (Andrews et al., 2015), symptomatic interventions. Furthermore, cognitive screening measures represent a relevant outcome within clinical trials targeting motor/non-motor features in HD patients (Stout et al., 2017).

However, as stressed by the Movement Disorders Society (MDS) in 2018 (Mestre et al., 2018), little consensus has been reached as to which cognitive screeners are the most adequate for use in this population, mostly due to the lack of disease-specific evidence on their psychometrics and diagnostics. Moreover, cognitive screening in HD patients is challenged by co-morbid behavioral manifestations and motor disability-related fatigue, which may alter the results of or hinder testing procedures (Papoutsi et al., 2014); (Snowden, 2017). Thereupon, an ideal cognitive screener for use in this population should be short-lived (≤ 15′) (Larner, 2017) and limitedly relying on tasks requiring fine movements, especially if timed (Carelli et al., 2021).

In such a framework, the Frontal Assessment Battery (FAB; Dubois et al., 2000) has been “suggested” by the MDS to screen for cognitive impairment in HD patients (Mestre et al., 2018): as having been designed for bedside evaluations, it is indeed brief (≤ 10′) and its motor-mediated tasks require the untimed execution of gross movements. Moreover, and most importantly, the FAB targets dysexecutive features, which predominantly characterize HD patients’ cognitive profile (Snowden, 2017).

However, the only study that has thus far employed the FAB in HD patients (Rodrigues et al., 2009), and based on which the MDS provided the aforementioned recommendation (Mestre et al., 2018), solely focused on its psychometrics (i.e., validity and reliability) and case–control discriminative power—this leading the MDS itself to prompt further investigations on the clinical usability of the screener in this population.

Given the above premises, this study aimed at providing, *via* receiver-operating characteristics (ROC) analyses, both intrinsic and post-test diagnostics of the FAB as to its capability to (1) discriminate healthy controls (HCs) from HD patients (i.e., case–control setting), and (2) detect cognitive impairment in HD patients (i.e., case-finding setting).

## Materials and methods

### Participants

Thirty-eight consecutive, genetically-diagnosed, motor-manifest HD outpatients (Reilmann et al., 2014) were recruited at IRCCS Istituto Auxologico Italiano, Milan, Italy and LIHR Foundation, Rome, Italy, along with 73 age-, education- and sex-matched healthy controls (HCs), between 2017 and 2021 (Table 1). Exclusion criteria were: (1) (HD-unrelated) neurological/psychiatric diagnoses; (2) severe general-medical conditions; (3) uncorrected hearing/vision deficits. This study was approved by the Ethics Committee of IRCCS Istituto Auxologico Italiano (I.D.: 2013_06_25); participants provided informed consent and data were treated according to current regulations.

**Table 1:** Participants’ demographic and clinical measures.

	HD	HCs	*p*
*N*	18/20	24/49	–
Sex (M/F)	16-Sep	39/62	0.135^a^
Age (y.)	60.18 ± 9.85 (27–78)	55.97 ± 14.45 (27–79)	0.113^b^
Education (y.)	13 ± 3.44 (8–17)	11.84 ± 3.55 (5–18)	0.104^b^
Disease duration (mo.)	103.58 ± 163.39 (12–852)	–	–
UHDRS-I	36.29 ± 15.62 (12–73)	–	–
UHDRS-II	128.74 ± 51.39 (51–272)		
UHDRS-III	18.27 ± 12.68 (0–44)		
UHDRS-IV	16.65 ± 6.41 (1–25)	–	–
UHDRS-V	76.22 ± 16.93 (30–100)	–	–
UHDRS-VI	8.14 ± 3.71 (1–13)	–	–
Shoulson-Fahn			
Stage 1	29.70%	–	–
Stage 2	35.10%	–	–
Stage 3	27%	–	–
Stage 4	8.10%	–	–
HTT triplets (*N*)	43.53 ± 3.82 (39–59)	–	–
FAB	13.5 ± 3.32 (7–18)	17.07 ± 1.54 (11–18)	< 0.001^c^
Below-cut-off scores^d^	44.70%	5.50%	–
MoCA	20.83 ± 5.6 (9–30)	–	–
Below-cut-off score^e^	41.70%	–	–

F = female; FAB=Frontal Assessment Battery; HCs = healthy controls; HD=Huntington’s disease; HTT = huntingtin; M = male; MoCA = Montreal Cognitive Assessment; UHDRS=Unified Huntington’s.

a *χ*^2^-statistic;

b *t*-statistic;

c Mann–Whitney *U*-statistic;

d Appollonio et al. (2005); and

e Aiello et al. (2022a,b,c).

### Materials

All participants were administered the FAB (Appollonio et al., 2005), with HD patients further undergoing the Montreal Cognitive Assessment (MoCA; Aiello et al., 2022c) and the Unified Huntington’s Disease Rating Scale (UHDRS) (Huntington Study Group, 1996)—the latter comprising six subscales, of which the first assesses motor function (UHDRS-I), the second attentive-/executive-based cognitive efficiency (UHDRS-II), the third dysexecutive, behavioral alterations (UHDRS-III), and the last three functional independence (UHDRS-IV/-V/-VI). Disease staging was derived *via* the Shoulson–Fhan system (Shoulson and Fahn, 1979). The FAB ranges 0–18 (administration time: ≤ 10′ (Dubois et al., 2000)) and comprises task assessing linguistically-mediated (i.e., concept formation and phonemic fluency) and motor-mediated executive functions (i.e., Luria’s sequence and sensitivity to interference), as well as inhibition (i.e., go-no-go and prehension behavior; Aiello et al., 2022b). The MoCA ranges 0–30 [administration time: ≤ 10′ (Mestre et al., 2018)] and assesses both instrumental (i.e., memory, language, visuo-spatial abilities, and orientation) and non-instrumental domains (i.e., attention and executive functions; Aiello et al., 2022c). The UHDRS-II [administration time up to 15′ (Mestre et al., 2018)] yields a total score that combines a phonemic fluency task, the Stroop Color and Word Test and the Symbol Digit Modalities Test (Huntington Study Group, 1996).

### Statistics

Within all ROC analyses, an age- and education-adjusted, below-cut-off score on the MoCA (Aiello et al., 2022c) was addressed as the positive outcome—pursuant to the 2018 MDS guidelines (Mestre et al., 2018) that provide a “suggested” level of recommendation towards the MoCA as a screener for cognitive impairment in HD patients, as well as to recent meta-analytic evidence further supporting its feasibility and clinimetric soundness in this population (Rosca and Simu, 2020).

As to case–control ROC analyses, the minimum sample sizes were estimated, according to Goksuluk et al. (2016), at *N* = 18 and *N* = 36 for HCs and HD patients, respectively, allocation ratio: (2), by addressing the following parameters: *α* = 0.05, 1–β = 0.8, AUC = 0.7. As to case-finding ROC analyses within the HD cohort, by forecasting, based on recent epidemiological evidence (Julayanont et al., 2020), a prevalence of cognitive impairment of 45%, the minimum sample sizes were estimated (Goksuluk et al., 2016) at *N* = 14 and *N* ≈ 17 for cognitively-impaired an -unimpaired HD patients, respectively (allocation ratio: 1.2) by addressing the following parameters: *α* = 0.05, 1–β = 0.8, AUC = 0.75.

Within both the aforementioned sets of analyses, sensitivity (Se), specificity (Sp), positive and negative predictive values (PPV; NPV) and likelihood ratios (LR^+^; LR^–^) were computed at the optimal cut-off identified *via* Youden’s *J* statistic.

Moreover, *via* the R package *pROC* (Robin et al., 2011), the diagnostic accuracy of the FAB was compared to that of the UHDRS-II by means of DeLong’s test for paired ROC curves.

Finally, the association between FAB scores and non-cognitive clinical measures, i.e., disease duration and UHDRS-I (assessing motor features), UHDRS-III (assessing behavioral features) and UHDRS-IV, -V and -VI scores (assessing functional outcomes)—were tested *via* Bonferroni-corrected Spearman’s correlations (as such disease-related variables did not distribute normally, i.e., skewness and kurtosis values ≥|1| and |3|, respectively; Kim, 2013). Moreover, the association between disease staging and FAB scores, which distributed normally (Kim, 2013)—was tested *via* a one-way ANOVA, followed by Bonferroni-corrected *post-hoc* tests.

Analyses were run with R 4.1^1^ and jamovi 2.3 (the jamovi project, 2022).

## Results

The completion rates for the FAB, MoCA and UHDRS-II in HD patients were of 100, 94.7% (*N* = 36) and 89.5% (*N* = 34), respectively. The prevalence of age- and education-adjusted, defective FAB scores (Appollonio et al., 2005) was of 44.7%. Fifteen out of 36 patients that managed to complete the MoCA were classified as cognitively impaired (41.7%).

Table 2 summarizes the results of ROC analyses within both case–control discrimination and case-finding scenarios.

**Table 2:** Diagnostics of the frontal assessment battery (FAB) in case–control and case-finding scenarios as addressed to HD.

	AUC	Cut-off	*J*	Se	Sp	PPV	NPV	LR^+^	LR^–^
HCs vs. HD									
Raw scores	0.86	≤ 15	0.56	0.68	0.88	0.74	0.84	5.6	0.36
Adjusted scores^a^	0.88	< 15.25	0.64	0.74	0.9	0.8	0.87	7.68	0.29
HD vs. HD with impaired MoCA^b^									
Raw scores	0.9	≤ 13	0.59	0.73	0.86	0.79	0.82	5.13	0.31
Adjusted scores^a^	0.91	< 14.35	0.71	1	0.71	0.71	1	3.5	0

FAB=Frontal Assessment Battery; HCs = healthy controls; HD=Huntington’s disease; MoCA = Montreal Cognitive Assessment; AUC = area under the curve; J = Youden’s index; Se = sensitivity; Sp = specificity; PPV = positive predictive value; NPV = negative predictive value; LR^+^ =positive likelihood ratio; LR^–^ = negative likelihood ratio.

a Appollonio et al. (2005).

b Aiello et al. (2022c).

FAB raw scores yielded, at the optimal cut-off (≤ 15; *J* = 0.56), high accuracy in discriminating HCs from HD patients (AUC = 0.86; SE = 0.04; CI 95% [0.79, 0.93]), with adequate both intrinsic (Se = 0.68; Sp = 0.88) and post-test properties (PPV = 0.74; NPV = 0.84; LR^+^ =5.6; LR^–^ = 0.36). When addressing age- and education-adjusted FAB scores (Appollonio et al., 2005), comparable, although slightly better, diagnostics were detected (AUC = 0.88; Se = 0.74; Sp = 0.9; PPV = 0.8; NPV = 0.87; LR^+^ =7.68; LR^–^ = 0.29) at the optimal cut-off (*J* = 0.64) of < 15.25.

As to its capability to identify HD patients with a below-cut-off MoCA score, raw FAB scores yielded, at an optimal cut-off of ≤ 13 (*J* = 0.59), excellent accuracy (AUC = 0.9; SE = 0.05; CI 95% [0.81, 1]), as well as adequate intrinsic (Se = 0.73; Sp = 0.86) and post-test properties (PPV = 0.79; NPV = 0.82; LR^+^ =5.13; LR^–^ = 0.31). According to such a cut-off, 42.1% of patients were classified as impaired on the FAB. When running the same analysis by addressing age- and education-adjusted FAB scores (Appollonio et al., 2005), slightly improved diagnostics were detected (AUC = 0.91; Se = 1; Sp = 0.71; PPV = 0.71; NPV = 1; LR^+^ =3.5; LR^–^ = 0) at the optimal cut-off (*J* = 0.71) of < 14.35—according to which, 60.5% of patients were classified as impaired.

Notably, when addressing patients who completed both the FAB and the UHDRS-II and comparing the raw scores from the two screeners as to their capability to discriminate those with an above- vs. below-cut-off score on the MoCA, AUC values did not differ (*z* = 0.74; *p* = 0.46)—despite that of the FAB being descriptively higher (AUC = 0.89; SE = 0.06; CI 95% [0.78, 0.1]) than that of the UHDRS-II (AUC = 0.85; SE = 0.07; CI 95% [0.72, 0.98]).

At *α*_adjusted_ = 0.008, raw FAB scores in HD patients proved to be associated with disease duration (*r_s_* = −0.46; *p* = 0.003) and both UHDRS-I (*r_s_* = −0.66; *p* < 0.001) and UHDRS-IV/-V/-IV scores (0.5 ≤ *r_s_* ≤ 0.54; *p* ≤ 0.002), but not with the UHDRS-III. Clinical staging affected raw FAB scores (*F*(3,33) = 3.6; *p* = 0.023), with such an effect being solely carried by raw FAB scores of patients in stage 3 (*M* = 12.2; SE = 0.91) being significantly lower (*p* = 0.035) than those of patients in stage 1 (*M* = 15.9; SE = 0.87).

## Discussion

The present study provides, for the first time, exhaustive evidence on the cross-sectional diagnostic soundness of the FAB in HD patients—thus further supporting the 2018 MDS recommendations as to the usefulness of such a screener in this population (Mestre et al., 2018). The FAB indeed yielded optimal diagnostic properties, when addressing both its raw and its demographically-adjusted scores (Appollonio et al., 2005), as to the discrimination between HCs from HD patients, performing even better at identifying cognitively-impaired HD patients.

This study is in line with Rodrigues et al. (2009) findings as to the fact that the FAB is able to discriminate HD patients from HCs—by nonetheless adding up to them as providing unprecedented information on the post-test diagnostic performance of this screener in case–control scenarios, which will help decision-making with respect to ruling-out/-in the presence of cognitive impairment based on FAB results. Most importantly, at variance with Rodrigues et al.’s (2009) report, the present one also provides both intrinsic and post-test diagnostics of the FAB in a case-finding setting, i.e., with regard to its capability to discriminate cognitively-impaired from -unimpaired HD patients. In this respect, given the not negligible difference between the cut-off derived when addressing raw (≤ 13) rather than demographically-adjusted FAB scores (< 14.35), it is advisable that Appollonio et al. (2005) original, age- and education-adjusted, normality cut-off of ≤ 13.4 be adopted in clinical practice and research as addressed to HD patients. In support of such a proposal, the present demographically-adjusted cut-off appeared to overestimate the occurrence of cognitive impairment when compared to Appollonio et al.’s (2005) one by 15.8%.

Interestingly, the FAB was comparable to the UHDRS-II as to the identification of cognitively-impaired HD patients, despite slightly outperforming it (89% vs. 85% of accuracy, respectively). With this regard, it is worth noting that 10.5% of patients did not manage to complete the UHDRS-II, whereas the FAB proved to be applicable to the whole cohort. Taken together, such findings suggest that, despite being widespread, the UHDRS-II may not necessarily represent the ideal choice to screen for cognitive impairment in HD patients. After all, the MDS itself pointed out that available clinimetric evidence on the UHDRS-II is currently limited—thus assigning it the same level of recommendation assigned to the FAB (Mestre et al., 2018). Indeed, the feasibility of the UHDRS-II as a cognitive screener in this population might be questioned as (1) taking up to 15′ to administer (Mestre et al., 2018), and (2) heavily relying on motor responses to be delivered as fast as possible. Moreover, as again highlighted by the MDS (Mestre et al., 2018), the UHDRS-II total score might not be clinically meaningful, since it corresponds to the mere sum of raw scores yielding from tests that do not fully overlap as to their target constructs. Thereupon, given the above considerations and, consequently, taking into account the trade-off between (1) administration time, (2) applicability, and (3) diagnostic properties, the FAB might be addressed, at least at a feasibility level, as more appropriate than the UHDRS-II when screening for cognitive impairment in HD patients. Nevertheless, it has to be stressed that, within the present investigation, the diagnostic accuracy of the FAB did not significantly differ from that of the UHDRS-II: hence, further studies are needed in order to determine whether the FAB could be actually regarded as superior to the UHDRS-II for screening aims in HD.

Finally, the finding of FAB scores being associated with both disease duration and motor/functional measures in HD patients (including clinical staging), besides confirming Rodrigues et al. (2009) results, supports the notion that this screener is overall sensitive to disease progression in this population. However, at the same time, this result warns on the fact that decreased FAB scores in HD patients might be to an extent confounded by an overall severer disease. Hence, future investigations should focus on determining the actual impact of motor-functional disabilities on FAB scores net of cognitive status. Moreover, the lack of association between the FAB and UHDRS-III scores provides divergent validity evidence for this screener, which would selectively capture cognitive, but not behavioral, dysexecutive features in HD patients.

The present study is of course not free of limitations. First, the sample size is relatively restricted, heterogeneous as to FAB performances (range = 7–18) and, most importantly, limited to motor-manifest HD patients—both these elements to an extent limiting the generalizability of the present findings. With this regard, it has to be highlighted that the diagnostics of the FAB still need to be tested on prodromal HD individuals, i.e., the pre-manifest population which is approaching the neurological onset of the disease: this would not only deliver pivotal evidence towards the usefulness of the FAB for detecting cognitive impairment in this population prior to the manifestation of motor signs, but also in the view of its adoption as an outcome measure within preventive clinical trials.

Second, a confounding effect of motor disabilities on FAB scores cannot be ruled out. However, this element is common to the UHDRS-II, and the high rate of applicability of the FAB, as compared to that of the UHDRS-II itself, appears to be reassuring towards such an issue. In this respect, it is nevertheless advisable that future studies focus on developing motor-free versions of such cognitive screeners (Poletti et al., 2016; Carelli et al., 2021).

Third, no subtest-level analyses have been herewith performed, at variance with Rodrigues et al.’s (2009) study: it is thus advisable that future investigations focus on testing the diagnostic performance of each FAB subtest (Aiello et al. 2022a,b,c).

Fourth, this work solely explored FAB diagnostics, thus not being exhaustive of the need, highlighted by the MDS (Mestre et al., 2018), to provide currently lacking psychometric properties for it in HD patients: further investigations on larger samples are thus advisable that focus on its validity and reliability.

Finally, it has to be noted that a specific *medium* has been herewith addressed, within ROC analyses, in order to operationalize cognitive impairment in HD patients, i.e., a below-cut-off MoCA score. Although the MoCA has received great support for use in this population (Rosca and Simu, 2020), further investigations are nevertheless needed to confirm the present findings against a different outcome, e.g., a second-level cognitive measure. In addition, one should bear in mind that the MoCA and the FAB do share some items (e.g., phonemic fluency and abstraction ones), this prompting future studies to focus on a reference measure that does not overlap with the FAB in any extent. However, in this respect, it should be noted that, within the present report, the MoCA yielded a prevalence of cognitive impairment that is consistent with previous epidemiological evidence in HD patients (Julayanont et al., 2020), as well as that the use of the MoCA as a reference for testing the diagnostics of the FAB has previously proved adequate in normotypical individuals (Aiello et al., 2022a)—both these elements supporting its adoption as an outcome variable within ROC analyses.

In conclusion, in HD patients, the FAB is featured by optimal diagnostic properties within both case–control and case-finding scenario and, by also taking into account previous disease-specific evidence on its feasibility, represents an optimal cognitive screener for clinical and research use.

## Data availability statement

The raw data supporting the conclusions of this article will be made available by the authors, without undue reservation.

## Ethics statement

The studies involving human participants were reviewed and approved by the Ethics Committee of IRCCS Istituto Auxologico Italiano (I.D.: 2013_06_25) and by the Institutional Review Board of LIRH Foundation (I.D.: 1.010721). The patients/participants provided their written informed consent to participate in this study.

## Author contributions

FS: conceptualization, data collection, drafting, and revision. EA: conceptualization, analyses, drafting, and revision. ST, LC, SM, and CC: data collection and revision. RF and AP: revision. SM, FV, VS, NT, BP, FS, and AC: conceptualization, resources, drafting, and revision. All authors contributed to the article and approved the submitted version.

## Funding

This research was funded by the Italian Ministry of Health to IRCCS Istituto Auxologico Italiano (Ricerca Corrente, project 23C923) and to IRCCS Casa Sollievo della Sofferenza (project 2101MH09). IRCCS Istituto Auxologico Italiano covered publication fees.

## Conflict of interest

VS received compensation for consulting services and/or speaking activities from AveXis, Cytokinetics, Italfarmaco, Liquidweb S.r.l., and Novartis Pharma AG, receives or has received research supports from the Italian Ministry of Health, AriSLA, and E-Rare Joint Transnational Call. He is in the Editorial Board of Amyotrophic Lateral Sclerosis and Frontotemporal Degeneration, European Neurology, American Journal of Neurodegenerative Diseases. BP and LC received compensation for consulting services and/or speaking activities from Liquidweb S.r.l. NT received compensation for consulting services from Amylyx Pharmaceuticals and Zambon Biotech SA. FS received compensation for consulting services and/or speaking activities from La Hoffman-Roche, Novartis, PTC Therapeutics, Wave Life Science, Prilenia.

## Publisher’s note

All claims expressed in this article are solely those of the authors and do not necessarily represent those of their affiliated organizations, or those of the publisher, the editors and the reviewers. Any product that may be evaluated in this article, or claim that may be made by its manufacturer, is not guaranteed or endorsed by the publisher.

## Footnotes

^1^ https://cran.r-project.org/
